# Hemoglobin level associates with survival in women from Appalachian Kentucky with uterine cervix cancer

**DOI:** 10.3389/fonc.2023.1132135

**Published:** 2023-07-06

**Authors:** Charles A. Kunos, Denise Fabian, Tricia Fredericks, Lauren Baldwin, Charles Dietrich, Rachel W. Miller, Frederick R. Ueland

**Affiliations:** ^1^ Department of Radiation Medicine, University of Kentucky, Lexington, KY, United States; ^2^ Department of Obstetrics and Gynecology, Division of Gynecologic Oncology, University of Kentucky, Lexington, KY, United States

**Keywords:** uterine cervix, cervical cancer, hemoglobin, survival, Appalachia

## Abstract

**Introduction:**

The purpose of this retrospective study was to determine the relationship between pretherapy hemoglobin levels and progression-free survival among women with uterine cervix cancer undergoing concurrent weekly cisplatin and radiotherapy followed by brachytherapy.

**Methods:**

Patients with advanced-stage II-IVA uterine cervix cancer were grouped by hemoglobin level (Hgb ≥ 12.0, 11.9-10.0, or < 10.0 g/dL). Endpoints were progression-free survival, overall survival, and local control.

**Results:**

Between 01/2001 and 07/2022, 168 patients contributed demographic, tumor, pretherapy hemoglobin, and outcome data with a median follow-up of 31 months. Progression-free survival at three years was 73% (95% confidence interval: 58%-84%), 71% (95% confidence interval: 56%-82%), and 62% (95% confidence interval: 44%-75%) for the Hgb ≥ 12.0, 11.9-10.0, or < 10.0 g/dL groups, respectfully (P < 0.001). In addition, pretherapy hemoglobin levels were significant with treatment outcome when included in a multivariate analysis of prognostic variables.

**Discussion:**

In conclusion, the difference in pretherapy hemoglobin level was prognostic of progression-free survival.

## Introduction

Radiotherapy with concurrent cisplatin-containing chemotherapy followed by brachytherapy is a standard for the medical care of patients with advanced-stage uterine cervix cancer (1, 2). Unfortunately, gains in local control and survival after concurrent radiochemotherapy have remained stagnant over the last two decades, particularly for those with advanced-stage disease in the pelvis (3).

In patients with uterine cervix cancer, anemia and tumor hypoxia are two clinical factors impacting strategies that intensify treatments aimed at improving local control and survival. A significant percentage of patients with uterine cervix cancer present with hemoglobin levels less than 12.0 g/dL (4, 5). Among American patients, 37 percent present with pretherapy hemoglobin levels of > 12.0 g/dL, 39 percent with 11.9-10.0 g/dL and 23 percent with < 10.0 g/dL (4). One randomized study examining the role of an erythrocyte stimulating agent (ESA) found that 58 percent of accrued patients received transfusion before or during radiochemotherapy for hemoglobin ¾ 10.0 g/dL (Gynecologic Oncology Group-0191, ref. 6). Most studies have shown pretherapy and on-treatment anemia predict for poorer local control and associate with shorter survival outcomes (4–7). The mechanism appears related to tumor hypoxia driving radiochemotherapy resistance while simultaneously stimulating angiogenesis (5). If these phenomena are causally related to poor treatment outcomes, a hemoglobin level < 10.0 g/dL and its resulting disrupted tumor oxygenation might associate strongly with progression-free survival (6). Despite this reasoning, treating physicians debate whether correcting anemia (< 10.0 g/dL) by administering blood transfusions provides enough clinical benefit in patients with uterine cervix cancer to justify the risk. Two studies examined local control of pelvic disease after administering blood transfusions, with better outcomes independently associated with on-treatment hemoglobin levels > 12.0 g/dL (4, 5).

Given this background, the current retrospective study was designed to investigate the association of pretherapy hemoglobin level and progression-free survival among uterine cervix cancer patients from Appalachian Kentucky and its surrounding communities.

## Materials and methods

### Study population

The University of Kentucky Institutional Review Board (Lexington, Kentucky, protocol #69443) approved this retrospective study. The study population was a sampling of women aged older than 18 years who were diagnosed with International Federation of Gynecology and Obstetrics (FIGO 1988) stages II to IVA uterine cervix cancer between January 2001 and July 2022 (**Table 1**). The study population resided in an urban manufacturing and rural agricultural region of central and eastern Kentucky (8). Patients in this study had either squamous, adenosquamous, or adenocarcinoma uterine cervix cancer histologies. Patients must not have had para-aortic lymphadenopathy by standard surgical or imaging criteria. Patients also must have undergone definitive intent external-beam radiotherapy with concurrent chemotherapy followed by brachytherapy regardless of whether they finished treatment or not (skewing the 25%-75% interquartile date range for reviewed patients to between February 2011 and October 2021). Patients were segregated into groups of pretherapy hemoglobin levels of ≥ 12.0, 11.9-10.0, or < 10.0g/dL. The University of Kentucky Markey Cancer Center provided all deidentified demographic, tumor, hemoglobin, and follow-up data. These data did not differentiate whether a patient received a blood transfusion to raise pretherapy or on-treatment hemoglobin level to greater than 10.0 g/dL.

**Table 1:** Demographic, tumor, and treatment characteristics of patient population (n = 168).

Characteristic	Number	Mean ± SD	%
Age, years	168	53.9 ± 13.6	
< 50	66		39
≥ 50	102		61
Race			
White	163		97
Black or African American	3		2
Other	2		1
Eastern Cooperative Oncology Group performance status			
0-1	122		73
2-3	46		27
Cell Type			
Squamous	132		79
Adenosquamous	4		2
Adenocarcinoma	32		19
Stage at diagnosis			
IIA	26		15
IIB	57		34
IIIA	8		5
IIIB	65		39
IVA	12		7
Tumor size, centimeter		5.4 ± 1.1	
≤ 4.0	23		14
> 4.0	145		86
Lymph node metastases			
Pelvic nodal metastases	69		41
Para-aortic nodal metastases	0		0
Duration of radiochemotherapy, days		61.5 ± 13.3	
< 60	73		43
≥ 60	95		57
Hemoglobin pretherapy, g/dL		10.7 ± 2.1	
≥ 12.0	54		32
11.9-10.0	55		33
< 10.0	59		35

SD, standard deviation of the mean.

### Treatment

External beam radiotherapy provided 45 Gy in 25 fractions using 3D conventional or intensity-modulated radiation therapy (IMRT) techniques. Intracavitary or interstitial brachytherapy involved either a prescription for low-dose-rate (40 Gy in 1 or 2 fractions) or high-dose-rate (27.5-30 Gy in 5 fractions) treatment. The overall radiation course duration was intended not to exceed 56 ± 3 days. Cisplatin (40 mg m^-2^, not to exceed 70 mg total per week) was given by vein once a week for six maximum cycles. Cisplatin dose modifications were permitted during external beam radiotherapy; however, there were no consistent guidelines for cisplatin dose modification among treating physicians during the study period.

### Statistical analyses

The primary endpoint of the current study was progression-free survival, with secondary endpoints of overall survival and local control. Progression was defined as posttherapy clinical or imaging evidence of persistent, recurrent, or metastatic uterine cervix cancer disease. Progression-free survival was determined by the interval between diagnosis to disease progression or death or to the date of the last contact for censored events (i.e., those alive with no evidence of disease). Overall survival was defined as the time from diagnosis to death or the date of the last contact for censored events (i.e., those alive regardless of disease status). Local control was considered successful if no disease developed in the irradiation portals. Local control was considered a failure if there was disease progression or persistence of disease posttherapy within the irradiation portal. Patients were generally followed quarterly for two years, semiannually for the next three years, and then annually until death.

**Figure 1:**
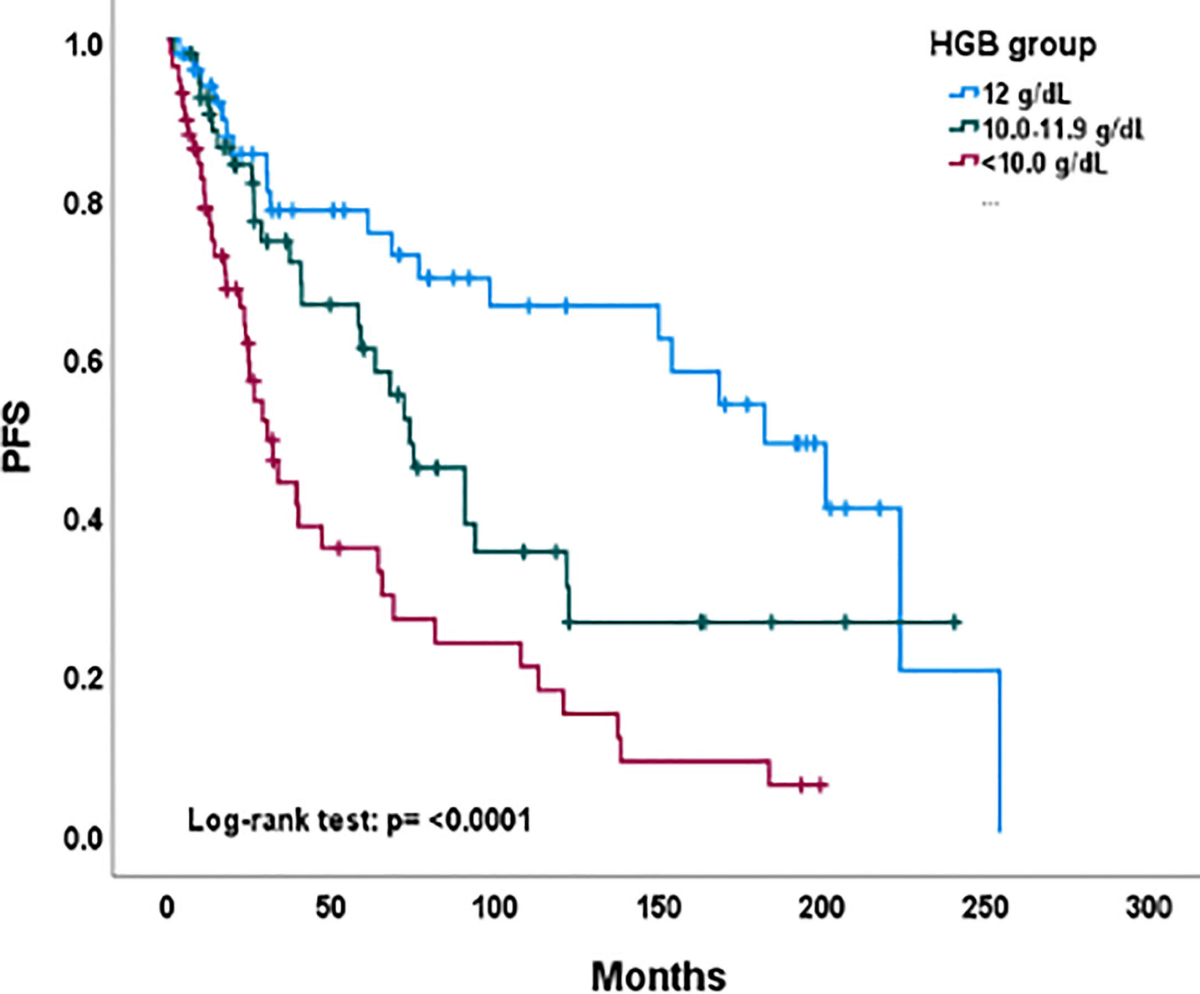
Progression-free survival by hemoglobin level group among all evaluable patients. PFS, progression-free survival; HGB, hemoglobin level.

Product-limit estimates for progression-free survival were calculated using the Kaplan-Meier method and differences by hemoglobin groups were compared by the log-rank test (**Figure 1**). The Cox proportional hazards model was performed to evaluate the impact of hemoglobin level on progression-free survival, adjusting for prognostic factors of age, tumor size, and duration of radiotherapy as continuous variables and race, performance status, disease stage, and cell type as categorical variables. The number of chemotherapy cycles received was not included in this analysis because this was not found to be a significant variable for outcome in our prior Appalachian uterine cervix cancer population study (8). Univariate analyses of these prognostic factors and pretherapy hemoglobin levels were evaluated by Student’s t-test (**Table 2**). A *P* value α less than 0.05 (two-sided) determined statistical significance. Data analyses used statistical software of Microsoft Excel (version 16.67) and SAS (version 9.4).

**Table 2:** Hemoglobin levels (g/dL) by demographic, tumor, and clinical characteristics.

Characteristic	Hemoglobin pretherapy	
	Mean ± SE	*P* value
Age, years		0.23
< 50	10.9 ± 0.04	
≥ 50	10.5 ± 0.02	
Race		0.60
White	10.7 ± 0.01	
Other	10.2 ± 0.49	
Eastern Cooperative Oncology Group performance status		0.06
0-1	10.9 ± 0.02	
2-3	10.2 ± 0.04	
Cell Type		0.35
Squamous	10.7 ± 0.02	
Other	10.4 ± 0.06	
Stage at diagnosis		0.01
II	11.1 ± 0.02	
III/IVA	10.2 ± 0.03	
Tumor size, centimeter		0.39
≤ 4.0	10.3 ± 0.09	
> 4.0	10.7 ± 0.01	
Duration of radiochemotherapy, days		0.61
< 60	10.8 ± 0.03	
≥ 60	10.6 ± 0.02	

Student’s t-test performed to compare the differences in hemoglobin level by two groups. SE, standard error of the mean.

## Results

### Characteristics of the study population

One hundred sixty-eight patients who received documented radiochemotherapy and had hemoglobin levels available for review were included in this retrospective study. The median follow-up was 31 months (25%-75% quartile: 14-88 months), and follow-up data were available for all 168 patients. Demographic, tumor, and treatment data are tabulated in **Table 1**. Of 168 patients, 155 (97%) were white (Caucasian), 122 (73%) had a Eastern Cooperative Oncology Group performance status of 0 or 1, 109 (65%) resided in rural Appalachian Kentucky, and 108 (64%) were smokers. The mean age was 54 ± 14 years. Patients had either stage II (49%), stage III (43%), or stage IVA (7%) uterine cervix cancer at diagnosis. One hundred thirty-two (79%) had a histopathological diagnosis of squamous cell carcinoma. The mean tumor size was 5.4 ± 1.1 cm. The median number of weekly cisplatin chemotherapy cycles was five (25%-75% quartile: 4-5 cycles). The mean duration of radiochemotherapy was 62 ± 13 days.

### Pretherapy hemoglobin level

Of 168 patients, all (100%) had pretherapy (baseline) hemoglobin levels available (**Table 1**). The mean pretherapy hemoglobin level was 10.7 ± 2.1 g/dL. Fifty-four (32%) had ≥ 12.0 g/dL, 55 (33%) had 11.9-10.0 g/dL, and 59 had < 10.0 g/dL. Pretherapy transfusion of blood to raise the hemoglobin level to greater than 10.0 g/dL was not reliably recorded in available data. A pretherapy hemoglobin level of < 10.0 g/dL was only significantly associated with stage at diagnosis (**Table 2**, *P* = 0.01).

### Outcome

As of December 2022, 80 (48%) patients were alive with no evidence of disease; 12 patients were alive with disease progression and 76 (45%) women had died. Nineteen (11%) patients had failed local control from radiochemotherapy. Thirty-three (20%) developed distant out-of-irradiation field recurrences. **Figure 1** depicts Kaplan-Meier estimates for progression-free survival by pretherapy hemoglobin level. Three-year progression-free survival estimates were 73 percent (95% confidence interval: 58%-84%), 71 percent (95% confidence interval: 56%-82%), and 62 percent (95% confidence interval: 44%-75%) for the hemoglobin levels of ≥ 12.0, 11.9-10.0, or < 10.0 g/dL groups, respectfully (*P* < 0.001). A multiple regression analysis of progression-free survival included age, stage, performance status, tumor size, and duration of radiochemotherapy. After adjusting for these five variables, pretherapy hemoglobin levels ≥ 12.0 (*P* < 0.001) and 11.9-10.0 (*P* = 0.04) were significant factors in progression-free survival. Age (*P* = 0.001) and stage (*P* < 0.001) were also significant in the multiple regression model.

## Discussion

Anemia and tumor hypoxia impact the likelihood of local disease control during the treatment of uterine cervix cancer. Correction of anemia before treatment possibly results in enhanced tumor oxygenation, and thus, increased radiochemosensitivity and desirable downstream disruption of molecular angiogenesis (5). While this phenomenon is not directly observable in our study, we did observe a 11 percent reduction in three-year progression-free survival estimate in our population when the pretherapy hemoglobin level was < 10.0 g/dL. Because there were no uniform guidelines regarding blood transfusion for anemia in our practice over the last two decades, we cannot comment on whether raising pretherapy hemoglobin level ≥ 10.0 g/dL improves uterine cervix cancer care outcomes. But as such, this retrospective study might prompt investigators to consider hemoglobin level < 10.0 g/dL more closely as an indication for blood transfusion between diagnosis and the start of radiochemotherapy.

In our univariate analyses, only higher advanced-stage III/IVA cases at diagnosis were more likely than stage II disease to have lower pretherapy hemoglobin levels. Prior investigators have reported poor performance status and large tumor size (> 4 cm) as associated with low hemoglobin levels in uterine cervix cancer patients before treatment (4, 5). The possible explanations for these associations are difficult to determine, but our prior studies suggest that impoverished patients tend to have a poorer performance status, seek medical care less frequently, and present with advanced-stage or bulky central pelvic disease (8). Differences in median household income, education level, and complex socioeconomic factors also adversely impact medical care, especially in rural regions like Appalachian Kentucky (8). A more sophisticated analysis is not possible with these data.

Prior investigators also indicated that pretherapy anemia was independently predictive of poor treatment outcomes (4, 6). More stringent analyses in their studies found that hemoglobin values on-treatment but not pretherapy were predictive of outcome when both variables were included in multivariate models (4). Our study does not capture on-treatment information; however, when adjusting for other prognostic factors, a pretherapy hemoglobin level < 10.0 g/dL remained a significant contributor to worse progression-free survival. The reasons underlying such an association of anemia and poor outcome remains elusive in patients with uterine cervix cancer. One alternate hypothesis offers the rationale that large hypervascular or advanced-stage tumors bleed more easily, manifesting as clinical anemia. Many consider low hemoglobin level < 10.0 g/dL an epiphenomenon associated with poor therapeutic outcome (4–7), and thus, transfusion intended to correct anemia would not be expected to provide clinical benefit. For example, Bishop and coauthors (9) published a retrospective study on over 2,400 advanced-stage uterine cervix cancer patients. The authors reviewed hemoglobin levels and disease outcomes following radiotherapy for uterine cervix cancer and concluded that only disease-specific survival (HR=1.49) was affected by hemoglobin levels < 10.0 g/dL. Multivariate analysis did not identify an impact on either central pelvic recurrence or distant metastases. Moreover, transfusion was associated with poor outcomes by all measures for the entire study cohort, though the mechanism for this remains unknown. A prospective evaluation is needed to understand the multitude of confounding factors related to anemia, cancer treatment, and disease outcomes. Our study findings do not resolve the controversial question of whether patients do poorly because they are anemic or because they have large hypoxic tumors that respond poorly to treatment and contribute to their anemia.

The most recent phase III cooperative group uterine cervix cancer study evaluating the effect of the ribonucleotide reductase inhibitor triapine in combination with standard radiochemotherapy used a ≥ 10.0 g/dL cutoff for trial eligibility (NCT02466971). Further research from prospective studies like this tracking pretherapy and on-treatment hemoglobin levels will shape good clinical practice guidelines for the pretherapy correction of anemia in uterine cervix cancer patients.

## Data availability statement

The raw data supporting the conclusions of this article will be made available by the authors, without undue reservation.

## Ethics statement

The studies involving human participants were reviewed and approved by University of Kentucky. Written informed consent for participation was not required for this study in accordance with the national legislation and the institutional requirements.

## Author contributions

All authors contributed to the article and approved the submitted version.
